# Drone delivery of an automated external defibrillator – a mixed method simulation study of bystander experience

**DOI:** 10.1186/s13049-019-0622-6

**Published:** 2019-04-08

**Authors:** J. Sanfridsson, J. Sparrevik, J. Hollenberg, P. Nordberg, T. Djärv, M. Ringh, L. Svensson, S. Forsberg, A. Nord, M. Andersson-Hagiwara, A. Claesson

**Affiliations:** 1 Department of Medicine, Centre for Resuscitation Science, Karolinska Institute, SE-17177 Stockholm, Sweden; 2 Västerbotten Emergency Medical Services, SE-90737 Umeå, Sweden; 3 Centre for Prehospital Research, Faculty of Caring Science, Work Life and Social Welfare, University of Borås, SE-, 501 90 Borås, Sweden

**Keywords:** Drone, UAV, AED, Defibrillator, Bystander experience, OHCA, CPR

## Abstract

**Background:**

Out-of-hospital cardiac arrest (OHCA) affects some 275,000 individuals in Europe each year. Time from collapse to defibrillation is essential for survival. As emergency medical services (EMS) response times in Sweden have increased, novel methods are needed to facilitate early treatment. Unmanned aerial vehicles (i.e. drones) have potential to deliver automated external defibrillators (AED). The aim of this simulation study was to explore bystanders’ experience of a simulated OHCA-situation where a drone delivers an AED and how the situation is affected by having one or two bystanders onsite.

**Methods:**

This explorative simulation study used a mixed methodology describing bystanders’ experiences of retrieving an AED delivered by a drone in simulated OHCA situations. Totally eight participants were divided in two groups of bystanders a) alone or b) in pairs and performed CPR on a manikin for 5 minutes after which an AED was delivered by a drone at 50 m from the location. Qualitative data from observations, interviews of participants and video recordings were analysed using content analysis alongside descriptive data on time delays during bystander interaction.

**Results:**

Three categories of bystander experiences emerged: 1) technique and preparedness, 2) support through conversation with the dispatcher, and 3) aid and decision-making. The main finding was that retrieval of an AED as delivered by a drone was experienced as safe and feasible for bystanders. None of the participants hesitated to retrieve the AED; instead they experienced it positive, helpful and felt relief upon AED-drone arrival and were able to retrieve and attach the AED to a manikin. Interacting with the AED-drone was perceived as less difficult than performing CPR or handling their own mobile phone during T-CPR. Single bystander simulation introduced a significant hands-off interval when retrieving the AED, a period lasting 94 s (range 75 s–110 s) with one participant compared to 0 s with two participants.

**Conclusion:**

The study shows that it made good sense for bystanders to interact with a drone in this simulated suspected OHCA. Bystanders experienced delivery of AED as safe and feasible. This has potential implications, and further studies on bystanders’ experiences in real cases of OHCA in which a drone delivers an AED are therefore necessary.

## Background

Out-of-hospital cardiac arrest (OHCA) affects some 275,000 individuals in Europe each year [1]. In Sweden in the year 2016, a total of 5132 cases of OHCA were reported to the Swedish registry for cardiopulmonary resuscitation (SRCR) with a 30-day survival rate of 11% and a majority of cases (93%) having a cerebral performance category (CPC) of 1–2 [2].

Shortening time from collapse to defibrillation is the most important factor for patient survival [3]. With time delays from collapse to defibrillation ranging 3–5 min, survival rates may reach 50–70% in witnessed cases of presumed cardiac etiology [4, 5].

EMS response time in Sweden has however increased in recent years from six minutes to ten minutes [2], and for various reasons, AED use has remained low [6]. Novel methods are therefore urgently needed that aim to reach OHCA patients with an AED at an earlier stage. Bystanders in close vicinity play an important role and can provide on-site defibrillation by using automated external defibrillators (AED) [7].

Unmanned aerial vehicles (UAV), i.e. drones, have the potential to be easily deployed in most geographical settings with low operational cost [8, 9].

Several studies have described this potential in geographic information system models (GIS) for deploying UAV i.e. drones to OHCA, showing promising results [10, 11]. In a recent GIS model the time reduction as compared to EMS was as high as 19 min [12]. This was later confirmed in 2016 in the same area using an AED drone for actual beyond visual line of sight (BVLOS) flights; the median time from dispatch to arrival of the drone was 5 min and 21 s whilst EMS delay was 22 min, saving a total of 16 min [13].

Although there is potential to deliver an AED, there is currently no data on bystander experiences of retrieving an AED as delivered by a drone. As drone technology emerges in the field of resuscitation, little is known of how this should be introduced in order to support bystanders in an OHCA situation in facilitating early defibrillation. Introduction of an additional resource may distract bystanders, interrupt CPR or create hands-off intervals when awaiting AED delivery. To our knowledge there are no studies on bystander interaction with AED-drone systems in which the AED actually comes into use.

The aim of this simulation study was therefore to explore bystander experience of an OHCA-situation where a drone delivers an AED and how the situation is affected by having either one or two bystanders onsite.

## Methods

In this simulation study, bystanders were presented to a simulated OHCA situation indoors with a manikin (Laerdal Resusci Anne™). A quasi-experiment [14] methodology approach was used combining qualitative data from observations, interviews of participants and video recordings alongside descriptive data on time delays during bystander interaction. The study was executed at a fire station in Western Sweden over 2 days in February 2018 with temperatures below zero Celsius outside. Participants were recruited from a senior citizen organization (PRO - the Swedish National Pensioners’ Organisation) since individuals who suffer from OHCA in Sweden have a median age of 71 years and the majority of OHCA take place at home [2] and CPR feasibly will be provided by a bystander of the same age. No form of compensation was awarded for participating in the study. Brief information about the study – a simulation study on suspected OHCA where participants would act as bystanders – was provided via email. Members were able to volunteer in the study and eight participants volunteered. None of the participants had any CPR or medical training in the last 20 years. Characteristics of study participants are presented in Table 1.

**Table 1:** Characteristics of study participants, (*n* = 8)

Variable	
Age years, median, (range)	75.5 (73–80)
Sex, female n (%)	4 (50.0)
CPR training the last 20 years n (%)	0 (0.0)
Prior medical training n (%)	0 (0.0)
Experience of real OHCA-situation n (%)	1 (12.5)
Participants using a smartphone n (%)	
**-**Female	4 (100.0)
**-**Male	0 (0.0)
Prior experience of interacting with a drone n (%)	0 (0.0)

**Fig. 1:**
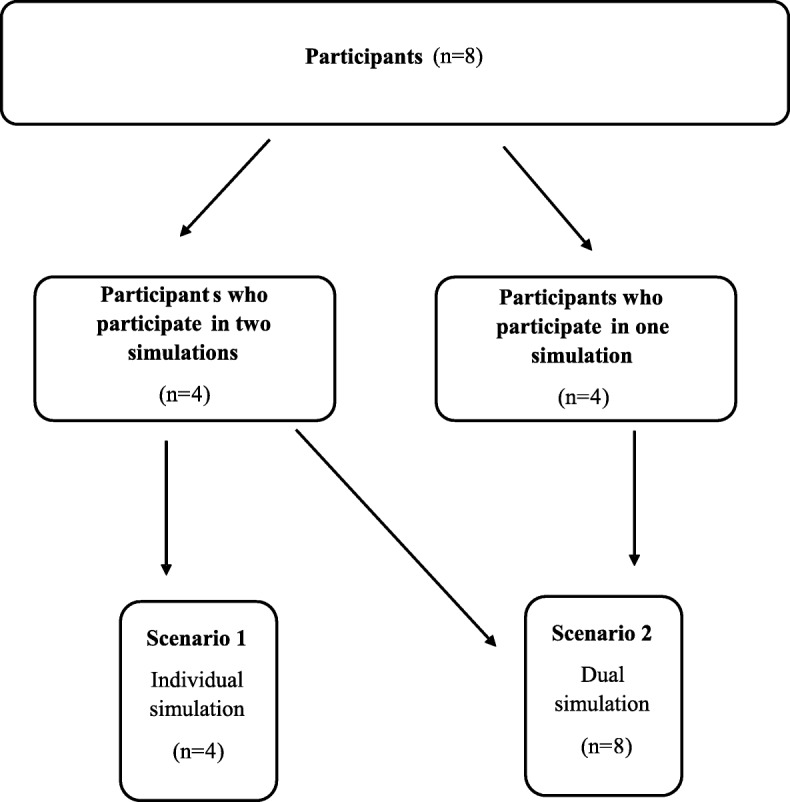
Flowchart of simulations. Two groups of bystanders, a) alone or b) in pairs, performed a simulated suspected OHCA-situation indoors at a fire station. Participants who performed in individual simulations also participated in dual simulations and acted in an assisting role. In their second simulation they only participated as assistant bystander. They had before the second simulation received clear directive that they were only allowed to be helpful with CPR if the first bystander asked them for help. They were not allowed to talk to the dispatcher, nor to fetch the AED or make any decision or suggestions, only to follow instructions

**Fig. 2:**
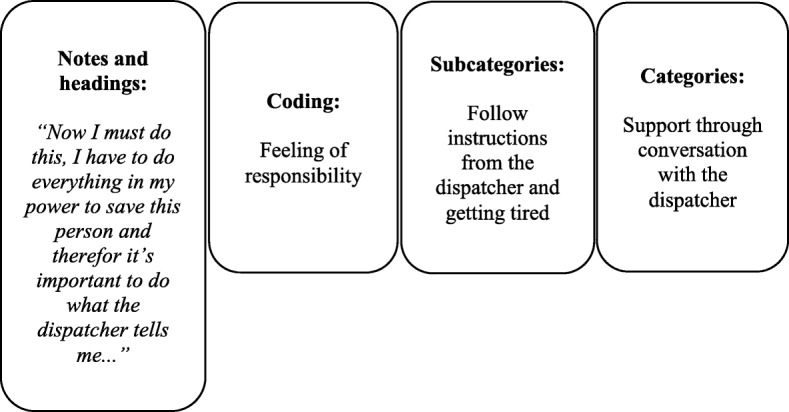
Example of inductive content analysis of data from the study. Qualitative data from open interviews with participants, observations and video recordings both from the drone and with a camera on site, were analysed using qualitative inductive content analysis. (26 Elo & Kyngäs 2007) Data was analysed and notes and headings were transcribed into coding until subcategories and categories emerged

**Fig. 3:**
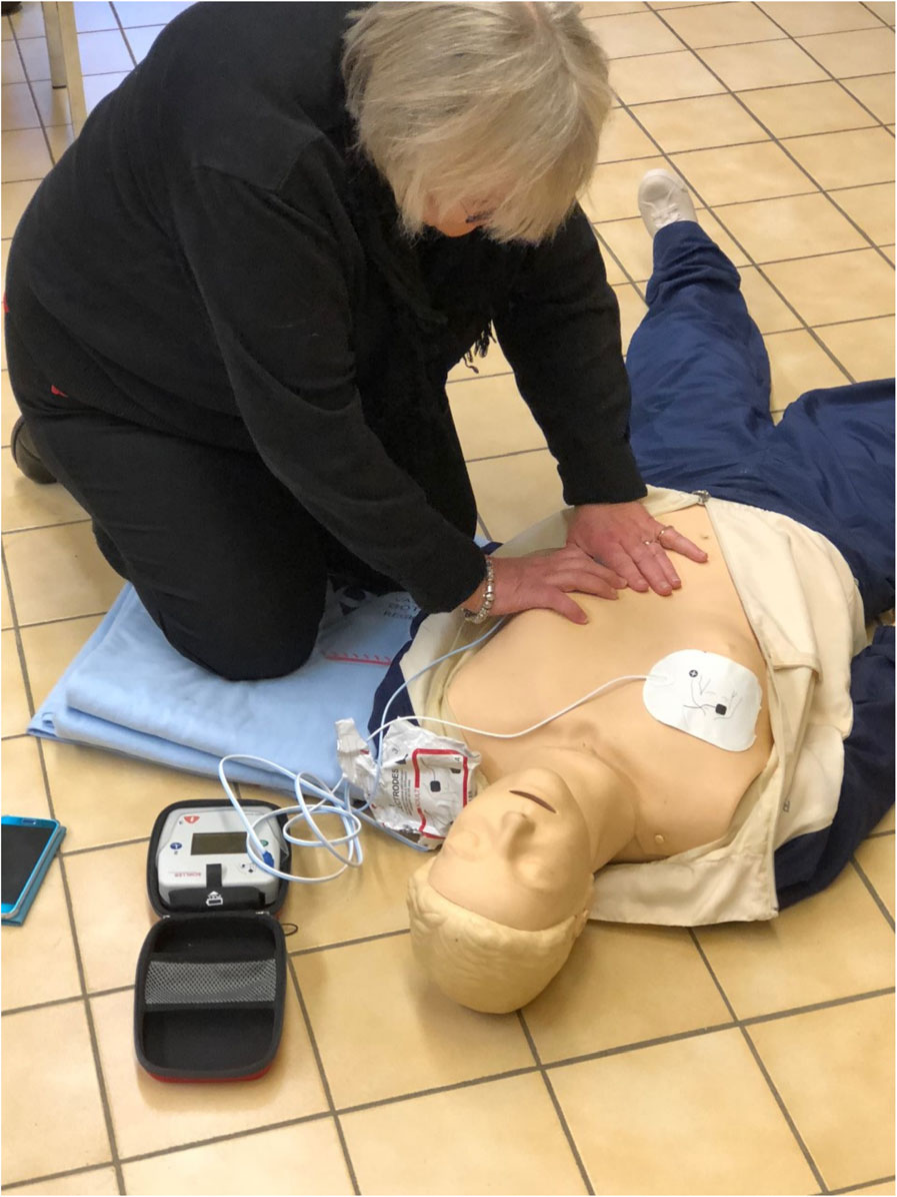
Photo of participant attaching AED-electrodes. Photo of participant attaching electrodes from AED (Schiller FRED easyport^TM^) to a manikin (Laerdal Resusci AnneTM) with a simulated suspected OHCA. This after retrieving the AED as delivered by drone (a modified DJI Inspire 1) 50 meters from the manikin

**Fig. 4:**
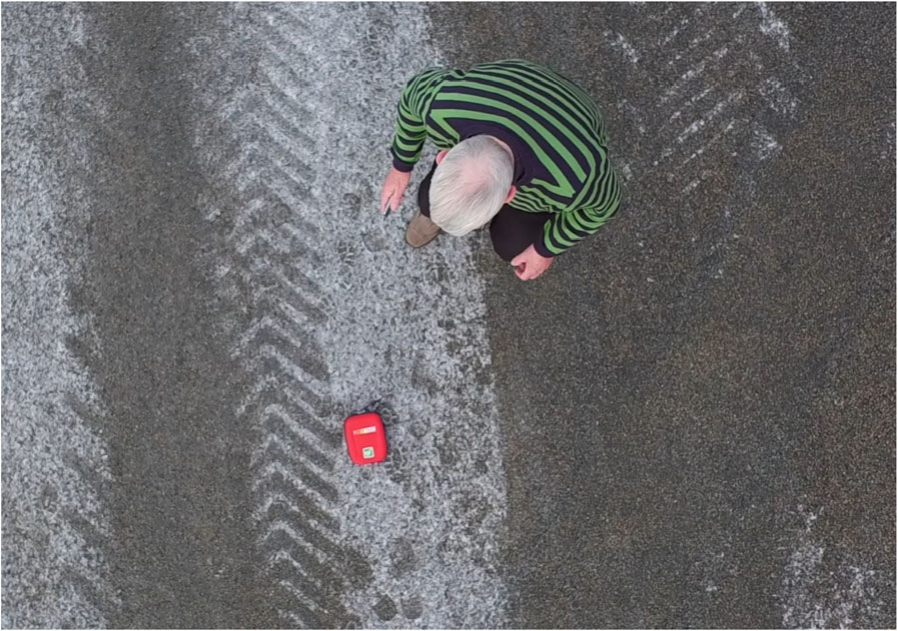
Photo of participant retrieving AED as delivered by a drone. Photo of participant retrieving AED (Schiller FRED easyportTM) after drone (a modified DJI Inspire 1) delivered AED 50 meters from the manikin and then hovered above at 10 m altitude, marking the location of the AED and provided livestream video to local dispatcher

**Fig. 5:**
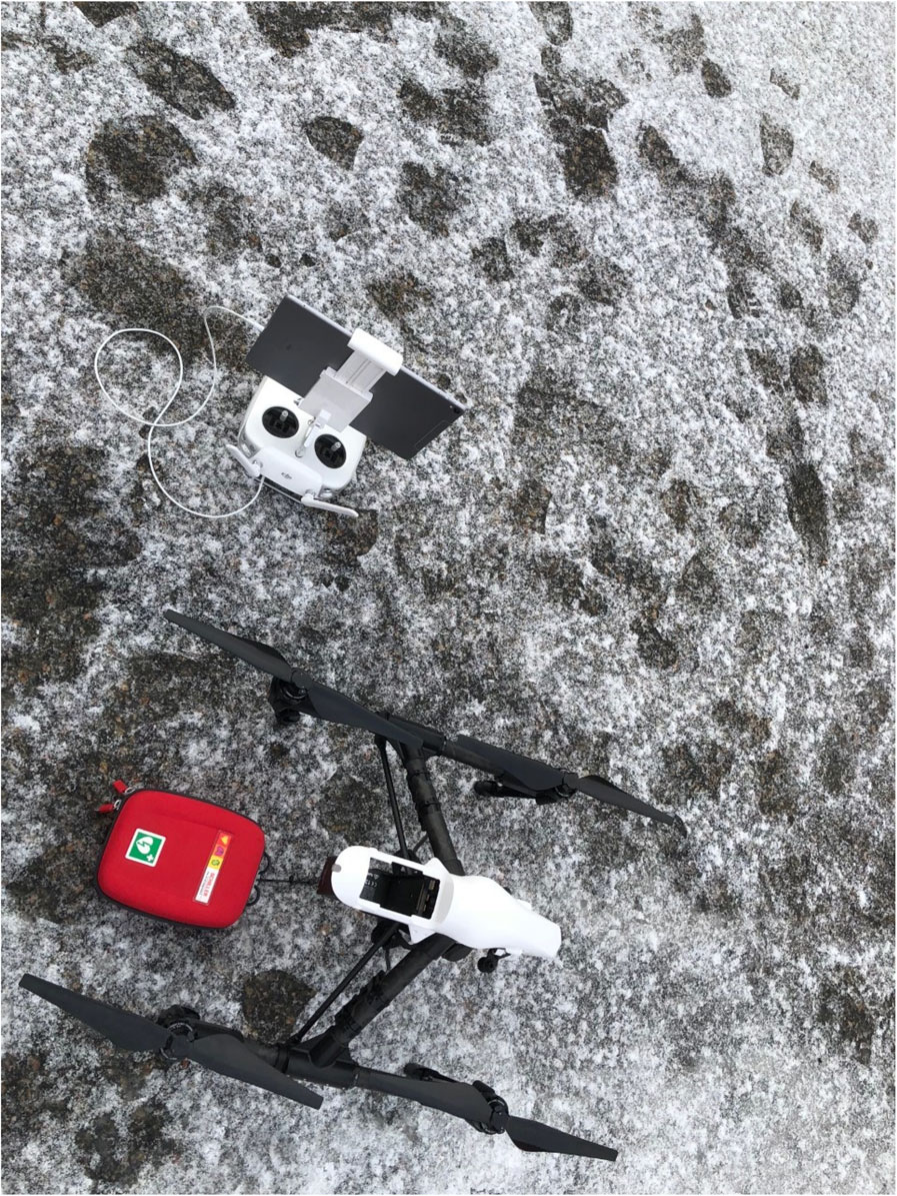
Photo of iPad (that provided live video streaming), drone and AED. Photo of iPad (version 3) that provided live video streaming, drone (a modified DJI Inspire 1) and AED (Schiller FRED easyportTM) that were used during simulations

Two groups of bystanders, a) alone *n* = 4 or b) in pairs *n* = 8, were told to interact in a simulated situation with a suspected OHCA indoors at a fire station. Participants were informed on the simulation day that a drone would deliver a defibrillator, and they were instructed to call 112 (emergency number in Sweden) for help from their own mobile phone to a local dispatcher and then follow instructions. The telephone number “112” was programmed onto participants’ own mobile phones which connected to a local dispatcher onsite that gave participants further instructions. The dispatcher followed medical index [15] for telephone assisted cardio-pulmonary resuscitation (T-CPR) instructions with CPR-algorithm 30 compressions and two ventilations. During the call information was provided that standard EMS (i.e. ambulance) and a drone was deployed. Based on previous data [13], the drone had a simulated flying time of 5 minutes. After 5 minutes of CPR, the dispatcher informed the bystander that an AED had been delivered on the ground by a drone at 50 m from the location. The drone, a modified DJI Inspire 1 (www.dji.com), landed, released an AED, (Schiller FRED easyport™) hovered at 10 m altitude, marking the place of the red AED-bag, and provided a live-video stream with visual feedback to the dispatcher. The simulations were completed when the participant attached electrodes from the AED onto the manikin. See Figs. 1, 2, 3, 4 and 5 for flowchart of simulations.

Open interviews with all participants were performed the same day of the simulation. Without being asked predetermined questions participants described their experiences during the simulation. Voice logs were recorded during interviews and transcribed into text. Alongside interviews and recording of interviews, live observations on set and video recordings both from the drone and from a camera on set were used during simulations. Both qualitative and quantitative data, i.e. time recordings (min:sec), from the simulations were compared to video data to increase validity. Qualitative data from open interviews with participants, observations and video recordings both from the drone and with a camera on site, were analysed using qualitative inductive content analysis [16]. Data was analysed and notes and headings were transcribed into coding until subcategories and categories emerged. An example of inductive content analysis of data from this study is presented in Fig. 2.

### Statistical analysis

For demographic data and time measurements, descriptive statistics were used. For categorical variables, cross tabulation was used. Due to the small sample size, all continuous variables are presented in median and range. To determine the time differences between a group’s single bystander and dual bystander, Mann-Whitney test was used. P level of < 0.05 was considered significant in all tests. All statistical analyses were performed using the statistical software program SPSS 21.0 (SPSS Inc., Chicago, IL). See Table 2.

**Table 2:** Time variables during simulated AED-drone interaction

Time variables	Single bystander median time (min:sec)	Dual bystanders median time (min:sec)	*p*
Bystander/s are introduced to a simulated OHCA – manikin			
1.Picks up the phone and start calling emergency operator,	0:13 (0:10–0:30)	0:05 (0:03–0:06)	0.02*
2. Picks up the phone and start calling emergency operator – until the emergency operator answer	0:32 (0:10–1:46)	0:18 (0:18–2:22)	0.88
3. Time for the emergency operator to recognise suspected cardiac arrest	1:04 (0:55–1:14)	1:31 (0:58–1:39)	0.56
4. Start CPR	2:21 (0:15–3:20)	2:25 (1:53–3:49)	0.47
Bystander is informed of AED-drone arrival at 5 min after dispatcher recognizes OHCA:			
5. Time for bystanders to retrieve AED 50 m away	1:34 (1:15–1:50)	2:06 (1:30–2:47)	0.24
6. Time for bystanders to attach AED to the patient	1:25 (0:40–1:28)	1:26 (0:47–1:21)	1.0
7. CPR hands-off time	1:34 (1:15–1:50)	0	0.01*
Total time delays:			
8. Emergency operator first answer the emergency call – until AED is attached to the patient	7:59 (6:55–8:18)	8:23 (7:28–8:34)	0.39
9. Total time from collapse until AED attached to the patient	9:47 (8:52–10:31)	10:27 (8:50–11:40)	0.56

* A *p*-level of <0.05 was regarded as significant

### Research ethics

This type of study is not within the boundaries of the ethic review act 2003:460 that regulates research involving humans in Sweden. The study was conducted in accordance with the requirements of the Helsinki Declaration [17].

## Results

A total of eight simulated OHCA-situations were performed with eight participants (50% female, aged 73–80 years). None of the participants had any prior CPR or medical training in the last 20 years, or prior experience with drones. All *n* = 4 women used smartphones but none of the men did. Only one participant had real life experience with OHCA, see Table 1.

### Bystanders experiences during drone delivery of AED

During the open interviews and observations, three main categories of bystanders’ experiences emerged in the qualitative analysis: 1) technique and preparedness, 2) support through conversation with the dispatcher, and 3) aid and decision-making.

### Technique and preparedness

Participants had a positive setting towards using drones to deliver an AED in suspected OHCA. *“It is good that technology has come this far and can be useful, instead of just playing with the drone….”*

In both observations and interviews, it was found that the use of their own mobile phone was the most difficult technical moment for the participants. Difficulties emerged in both being able to call the dispatcher and to activate the speakerphone during the simulated OHCA-situation. *“In the beginning I had trouble with the mobile phone which made me very nervous….”*

Difficulties regarding handling the AED also emerged during observation and primarily concerned attachment and placement of electrodes, however with support from the dispatcher, attaching AED electrodes were viable. Difficulties regarding handling the AED differed among participant*. “It felt good and I had no trouble attaching the electrodes. The dispatcher gave me good instructions and the pictures on the electrodes showed me how to place the electrodes….”*

During the interviews it also emerged that the feeling of stress about handling technique differed between participants. Participants who expressed a more positive approach towards technique (mobile phone, drones and AEDs) was observed to perform better in the simulation. One participant expressed during the interview, *“It is amazing and easy with todays mobile phone. It would have been much more difficult before without speakerphone….”* another participant expressed about drones, *“This can save lives’….”*

Compliance with dispatcher instructions differed between participants. Those participants who expressed in the interview that they felt stressed during the simulation was observed to have harder time performing correctly in all tasks they were given. For example, compliance to CPR-instructions was lower and information and instructions from the dispatcher more often got misinterpreted. This did not occur to the same extent among those participants who did not express the feeling of stress during interviews. Regardless of these findings, all participants reacted with determination to retrieve the AED when the drone arrived and none of the participant hesitated or misinterpreted instructions when the dispatcher asked them to retrieve the AED as delivered by a drone. During interviews, one participant expressed about the dispatcher’s instructions to retrieve the AED: *“Now I must hurry, because I need help….”*

#### Support through conversation with the dispatcher

All participants expressed during the interviews that interacting with the dispatcher gave them a sense of security and support and made it easier handle the situation and perform the tasks that were given, *“Since the dispatcher explained so well, the situation seemed less uncomfortable….”*

During the interviews it became clear that communication with the dispatcher played a crucial role in how the participant managed to handle the situation, *“Now I must do this, I have to do everything in my power to save this person and therefor it’s important to do what the dispatcher tells me....”*

The participants expressed during the interviews that the dispatcher made them more confident about retrieving the AED, *“The dispatcher told me it was safe to retrieve the AED, so it didn’t feel scary at all.”*

It emerged in observations and in video recordings that long instructive sentences during T-CPR regarding the drone affected CPR in a negative way. Participants stopped compressions to listen to the dispatcher when too much information was given at the same time. Conversely, short encouraging sentences like *“you’re doing a good job”* or *“help is on its way”* had an observed positive effect on CPR-compressions.

#### Aid and decision making

In interviews the participants expressed concern about finding the AED fast enough after it was delivered by the drone and also about having direct physical contact with the drone. When participants saw the drone hovering above the ground, thus marking the location of the AED, they expressed during the interviews that they reacted positively as this made it easier to find the right location. *“I didn’t find it scary at all to retrieve the AED. As soon as I saw the AED I forgot about the drone….”*

All participants also reacted positively to the red AED bag which made it easier to find the AED on the ground. Participants also expressed a wish for the drone to have headlights in order to mark the location of the AED. During simulations, observations showed that none of the participants hesitated as they approached the drone to retrieve the AED. Instead they confirmed during interviews that they felt a sense of relief when the drone arrived. *“It felt good, calming in some way, and I felt that now I will get help. Even if there were no person who came, it made no difference. The important thing was that someone or something came to help me….”* During the interviews participants also expressed about drone arrival, *“It felt good to get help…”, “perfect…”, “finally….”*

Participants were instructed to retrieve the AED and therefore during this time CPR was deprioritised when only one bystander was involved in the simulation. The following thoughts were expressed by a lone-bystander participant during interviews: *“It felt good that an AED had arrived, maybe an AED could help better than the CPR I had tried to perform for so long…”.*

During the interviews participants expressed a concern about leaving the person with OHCA on the floor without CPR while retrieving the AED. Aside from this concern, a participant came to the conclusion that they needed more help because they were afraid they could not maintain the strength needed to continue CPR compressions. Another participant expressed, *“If you would have been two participants, one could have stayed and continue CPR while the other one retrieved the AED. It didn’t feel a 100% right to live the person alone on the floor….”*

Participants who performed the simulation in pairs all expressed that it felt safer and more manageable not being alone, *“I thought it was positive being two in this situation because it made me feel more secure and we could help each other to perform CPR. I didn’t feel so exposed in the pair-simulation as I feel when I performed the simulation alone.…”.*

### Interaction with dispatcher and the AED delivering drone

The median time for bystanders to initiate a call to the EMDC after finding the manikin on the floor was 13 s (range 10–30 s.) with one bystander and 5 s (range 3–6 s.) with two bystanders on site (*p* = 0.02). With one bystander on site, time interval retrieving the AED, as delivered by the drone at 50 m from the manikin, as well as hands-off time (time when no bystanders are beside the patient and can perform CPR) was 94 s (range 75–110 s.). With two bystanders on site, the time for one of the bystanders to retrieve the AED was 126 s (range 90–167 s.) (*p* = 0.01). The other bystander continued CPR, and therefore hands-off time (no CPR) was 0 s. No differences were seen in any other time variable. See Table 2.

## Discussion

The aim of this simulation study was to explore bystanders experience of a simulated OHCA-situation where a drone delivers an AED and how the situation is affected by being one or two bystanders onsite, a mixed methodology was used.

The main finding was that it made good sense for the bystanders to interact with a drone in this simulated suspected OHCA and that retrieval of an AED as delivered by a drone was experienced as safe and feasible for bystanders.

### Technique and preparedness

Bystanders experienced it positive, helpful and felt relief regarding AED-drone arrival and were able to retrieve and attach the AED to a manikin. Interacting with the AED-drone was perceived as less difficult than performing CPR or handling the mobile phone during T-CPR.

Some of the participants expressed during the interviews, that they felt stressed about handling the technical moments in the simulation. Observations showed that those participants also had more difficulties performing the tasks that were given, had a harder time handling technique such as mobile phone and placement of AED-electrodes and more often misinterpreted information and instructions from the dispatcher. Earlier studies on OHCA- and CPR-situations highlight that bystanders feel a sense of panic and how this inhibits the ability to follow instructions from the dispatcher [18]. Further studies show that bystanders experience OHCA situations as overwhelming and too hard to handle and this can make them paralysed and unable to perform CPR [19]. The ability to follow instructions from the dispatcher affects the result of the treatment. Time is of the essence in OHCA situations and has a direct impact on the patient’s chance of survival [2].

None of the participants had any prior CPR training the last 20 years to rely on. Earlier studies show that CPR training rarely reaches elderly people [20] a demographic that is likely to be present in OHCA-situations. CPR training should both be widely distributed in society and focused toward cohorts of people likely to be onsite in an OHCA situation.

### Support through conversation with dispatcher

Participants expressed during the interviews that the dispatcher made them feel safe and had a positive effect on the ability to perform necessary tasks. Thorén et al. also show in their study how dispatchers play an important role in how the bystander feels during an OHCA situation and that encouragement from dispatchers has a positive effect on performing CPR [18]. Delivery of an AED using a drone seems feasible, and support from the dispatcher will probably have a large impact on the outcome. During the study we observed that decreased bystander compliance with CPR instructions seemed to correlate with increased sentence length in dispatcher instructions. Further studies will be needed on drones delivering AED, and dispatchers will need training in how to inform bystanders about the drone delivering an AED during OHCA situations so that CPR does not get interrupted. An updated T-CPR protocol that includes drone delivery of an AED will be necessary in further studies.

### Aid and decision making

None of the participants hesitated when the dispatcher instructed them to retrieve the AED as delivered by a drone. Instead they reacted with determination to help the person with a suspected OHCA. Participants experienced AED arrival as a relief and felt that help arrived with the drone. Participants had no trouble retrieving the AED and experiences it positive when the drone hovered above and marked the location of the AED. The red colour of the AED bag also reportedly facilitated locating the AED more easily. Participants also expressed a desire for headlights on the drone marking the location of the AED and flashing lights on the AED to facilitate spotting the AED even more. Live video stream from the drone in real time to the dispatcher could perhaps enhance dispatchers’ understanding of the situation and make retrieval of the AED safer and more efficient. Delivering and releasing the AED on the ground before bystanders retrieve the AED, may prohibit direct contact with the drone thus making the delivery safer. Another possible way to deliver an AED may be to hover and winch the AED down and release it on the grown so that the drone stays in the air at all time, marking the place of the AED and prohibiting direct contact with the bystander. However, optimal ways to deliver an AED using a drone, needs to be tested more thoroughly in a real-life setting.

### Interaction with dispatcher and the AED delivering drone

Another finding was that single-bystander simulation introduced a significant hands-off interval when retrieving the AED 50 m from the patient. Median hands-off time introduced with one participant was 94 s. Participants in this study having a median age of 75.5 years and the fact that simulations took place during winter in cold conditions probably both affect hands-off intervals. Hands-off time during better weather conditions and with younger bystanders may be shorter; however, the simulations are representative of a real OHCA situation in Sweden, considering both weather and presumable bystanders on site. In a real-life situation, median distance to landing place and AED pick-up may be longer. In follow-up study, different ways of AED-delivery will be tested, to optimize AED pick-up. Earlier studies show that patient’s chance of survival after treatment with an AED decreases when CPR is not sustained. The longer “hands-off” time, the less likely it is for AED treatment to work [21] as patient’s chance for survival decreases by 10% for each minute without CPR [2].

To avoid long hands-off time, this system should only be used in situations when there is more than one bystander. Furthermore, with EMS response time in Sweden having increased in recent years from 6 minutes to 10 minutes [2], and due to the fact that quality of CPR compressions wanes after 1 minute and continues to decrease for each subsequent minute [22], it may be difficult for one bystander to perform high-quality CPR while waiting for the ambulance. Further research is needed to evaluate if an AED, as delivered by a drone, can be an alternative in situations with only one bystander, although it will result in CPR-interruption.

Public access defibrillation (PAD) before emergency medical services (EMS) arrival has been shown to increase survival [23] and mobile positioning systems have been introduced in Sweden to shorten delays by dispatching volunteers (SMS-lifesavers) to perform CPR and fetch public AEDs. However, the study suggests relatively long distances to nearby AED with a median distance of 1280 m (IQR 748–1776 m) [24], and the majority of public AEDs are placed in workplaces and shops [6], making the AED unavailable at all hours. A drone delivering an AED could benefit all cases of OHCA regardless of the number of bystanders onsite, the location of the OHCA or the time of day that the OHCA occurs, thus increasing the chance of early defibrillation. To optimize the prerequisites for survival of OHCA, integrating AED-drone system in SMS-lifesavers application may be a solution and could provide SMS-lifesaver that arrive to perform CPR and retrieve AED as delivered by a drone. Since drone landing places are heterogenous, the SMS-app technology may require direct communication with the dispatcher.

### Limitations and strength

This explorative study presents with several relevant and important limitations. Tests were performed in a strictly controlled and simulated setting with few study participants. Although following standard T-CPR protocol, a Laerdal manikin and a locally placed dispatcher were used. The drone was able to deliver the AED on flat ground in daylight at 50 m from the location at all times, conditions that may differ heavily from real life OHCA situations. It is also likely that a real OHCA situation comes with a different level of stress compared to a simulated OHCA. Participants who performed in two simulations were only allowed to act as assistant bystander in the dual simulation and follow instructions regarding CPR from the first bystander. They were not allowed to make any decisions or suggestions nor retrieve or attach the AED or talk to the dispatcher. However, you could argue that the first simulation gave them prior experience.

Open interviews without predetermined questions may have shifted focus from drone delivery to other parts of the simulation.

One of the strengths of the study was that participants represented individuals likely to become bystanders in OHCA situations. Both their age and the fact that they had no prior experience with drones, CPR or medical training increase the validity of the study. Simulations also took place during weather conditions that are representative and common for Sweden.

## Conclusions

The study shows that it made good sense for bystanders to interact with a drone in this simulated suspected OHCA. Bystanders experienced drone delivery of an AED as safe and feasible. This has potential implications, and further studies on bystanders’ experiences in real cases of OHCA in which a drone delivers an AED are therefore necessary.

## Abbreviations

AED: Automated external defibrillator
BVLOS: Beyond visual line of sight
CPC: Cerebral performance category
CPR: Cardio-pulmonary resuscitation
EMS: Emergency medical services
GIS: Geographical information system
OHCA: Out of hospital cardiac arrest
PAD: Public access defibrillation
SRCR: Swedish registry for cardiopulmonary resuscitation
T-CPR: Telephone assisted cardio-pulmonary resuscitation
UAV: Unmanned aerial vehicle
